# Regression Modeling and Meta-Analysis of Diagnostic Accuracy of SNP-Based Pathogenicity Detection Tools for *UGT1A1* Gene Mutation

**DOI:** 10.1155/2013/546909

**Published:** 2013-08-13

**Authors:** Fakher Rahim, Hamid Galehdari, Javad Mohammadi-asl, Najmaldin Saki

**Affiliations:** ^1^Golestan Blv. Toxicology Research Center, Ahvaz Jundishapur University of Medical Sciences, Ahvaz, Iran; ^2^Genetic Department, Faculty of Science, Shahid Chamran University, Ahvaz, Iran; ^3^Department of Medical Genetics, Ahvaz Jundishapur University of Medical Sciences, Ahvaz, Iran; ^4^Research Center of Thalassemia & Hemoglobinopathy, Ahvaz Jundishapur University of Medical Sciences, Ahvaz, Iran

## Abstract

*Aims*. This review summarized all available evidence on the accuracy of SNP-based pathogenicity detection tools and introduced regression model based on functional scores, mutation score, and genomic variation degree. *Materials and Methods*. A comprehensive search was performed to find all mutations related to Crigler-Najjar syndrome. The pathogenicity prediction was done using SNP-based pathogenicity detection tools including SIFT, PHD-SNP, PolyPhen2, fathmm, Provean, and Mutpred. Overall, 59 different SNPs related to missense mutations in the *UGT1A1* gene, were reviewed. *Results*. Comparing the diagnostic OR, our model showed high detection potential (diagnostic OR: 16.71, 95% CI: 3.38–82.69). The highest MCC and ACC belonged to our suggested model (46.8% and 73.3%), followed by SIFT (34.19% and 62.71%). The AUC analysis showed a significance overall performance of our suggested model compared to the selected SNP-based pathogenicity detection tool (*P* = 0.046). *Conclusion*. Our suggested model is comparable to the well-established SNP-based pathogenicity detection tools that can appropriately reflect the role of a disease-associated SNP in both local and global structures. Although the accuracy of our suggested model is not relatively high, the functional impact of the pathogenic mutations is highlighted at the protein level, which improves the understanding of the molecular basis of mutation pathogenesis.

## 1. Introduction

Crigler-Najjar syndrome (CNS) (MIM nos. 218800, 606785) type I and type II are inherited as autosomal recessive conditions that is resulted from mutations in the *UGT1A1 *gene (*UGT1A1*; MIM nos. 191740) [1–4]. Type I is characterized by almost complete absence of *UGT1A1* enzyme activity, and these patients are refractory to phenobarbital treatment, while type II is a less severe form of deficiency [5, 6]. Patients with CNS are at permanent risk of developing severe neurologic complications such as hearing problems, mental retardation, and choreoathetosis due to severe unconjugated hyperbilirubinemia [7]. It is well known that *UGT1A1* is expressed specifically in the liver and that it is difficult to perform an expression analysis directly on the patients by invasive liver biopsy but to state that the mutation causes inactivation of the enzyme you could perform an in vitro functional study by cloning the mutated cDNA of *UGT1A1* in an expression vector. The constructs could be transfected in hepatic cell lines as HepG2 or HUH7. The expression analysis on these cells overexpressing the mutated forms of *UGT1A1* will allow you to finally demonstrate the inactivation of the enzyme [8]. The *UGT1A1 *gene comprises five consecutive exons located on chromosome 2q37 by which complete or partial inactivation of any exon causes CNS. Single variations in deoxyribonucleic acid (DNA) base pairs responsible for protein, called coding which is single nucleotides polymorphism (SNP), leads to changes in amino acids that ultimately affect the protein structure and function. Different such types of SNPs include, missense mutations, nonsense, silent mutations, and splice-site mutations. The majority of missense mutations leads to considerable variation in the protein structure and function, causing the disease symptoms. Data about nonsynonymous SNPs exists in public repositories such as SWISSPROT [9], dbSNP [10], and HGVBASE [11]. 

Genetic methods including the detection of genes linked to the disease phenotypes and the identification of aberrant functions of these genes have, in recent years, provided worthy understanding into the biological foundations of genetic mutation [12]. The present review summarized all available evidence on the accuracy of SNP-based pathogenicity detection tools and introducing regression model based on different scores including functional scores, mutation score, and genomic variation degree and compared the results to the published clinical result.

## 2. Materials and Methods

### 2.1. SNP Data Sources and Collection

An inclusive search was done to find all CNS-related mutations. The major data repositories, including HGMD, dbSNP, SNPdbe, and Ensembl, were reviewed. All CNS-related mutations were extracted and double checked for duplicated queries and then tabulated (Table 1). 

### 2.2. Inclusion Criteria

Only *UGT1A1*-gene-related missense mutations were included.

### 2.3. Exclusion Criteria

Other types of mutation such as synonymous or nonsense were excluded.

### 2.4. Data Extraction

The pathogenicity prediction was done using SNP-based detection tools including SIFT [13], PHD-SNP [14], PolyPhen2 [15], fathmm [16], Provean [17], and Mutpred [18]. Then a regression model was designed using functional scores, mutation score, and genomic variation degree. For each SNP-based pathogenicity detection tool and our regression model, we extracted a 2 × 2 table including positive prediction of the disease (True Positive, TP), negative prediction as neutral (true negative, TN), positive prediction in nondisease (false positive, FP), and negative prediction in disease (false negative, FN). In order to assess the phenotypic characterization and clinical features of the disease of interest, we searched databases, including SWISSPROT [9], dbSNP [10], Ensembl [19], OMIM [20], DECIPHER [21], and HGVBASE [11]. Furthermore, we compared the results of SNP-based pathogenicity detection tools with the results of phenotypic description tools. Then we calculated the diagnostic odds ratio (diagnostic OR), which is a single indicator of test performance and varies between 0 and infinity [22]. 

### 2.5. Statistical Analysis

All the analyses were done by SPSS 16.0. A regression model was designed using three categories, including functional score [23], structural score (GV, genomic variation score) [24], and conservation score [25]. Each SNP-based pathogenicity detection tool was compared by the reference values using logistic regression. The sensitivity (Sn), specificity (Sp), accuracy (ACC), diagnostic OR, and Matthew's correlation coefficient (MCC) were calculated using the following formula:

(1) Sensitivity(Sn)=TPTP+FN,Specificity(Sp)=TNTN+FP,Accuracy=TP+TNTP+FP+TN+FN,Diagnostic  OR=Sn/1−Sn1−Sp/Sp,MCC=(TP×TN)−(FP×FN)(TP+FP)(TP+FN)(TN+FN)(TN+FP).

The metadisk was used to calculate individual and pooled diagnostic OR, sensitivity, specificity, negative likelihood ratio, and positive likelihood ratio [26]. We also compared the AUC (area under curve), which is a popular index of the overall performance of a test, using the summary receiver operating characteristic (SROC) curve [27]. 

## 3. Results 

**Figure 1:**
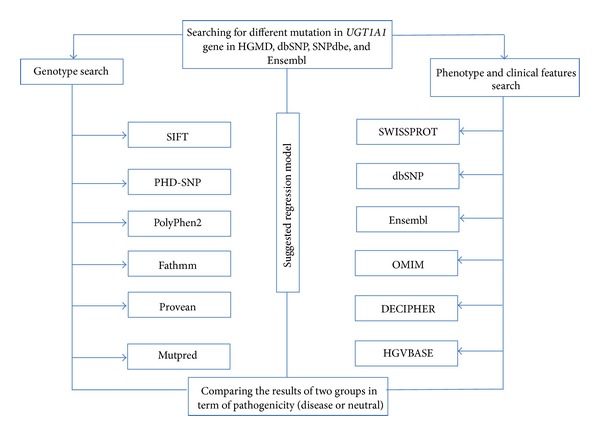
Flowchart of searching for SNPs.

**Figure 2:**
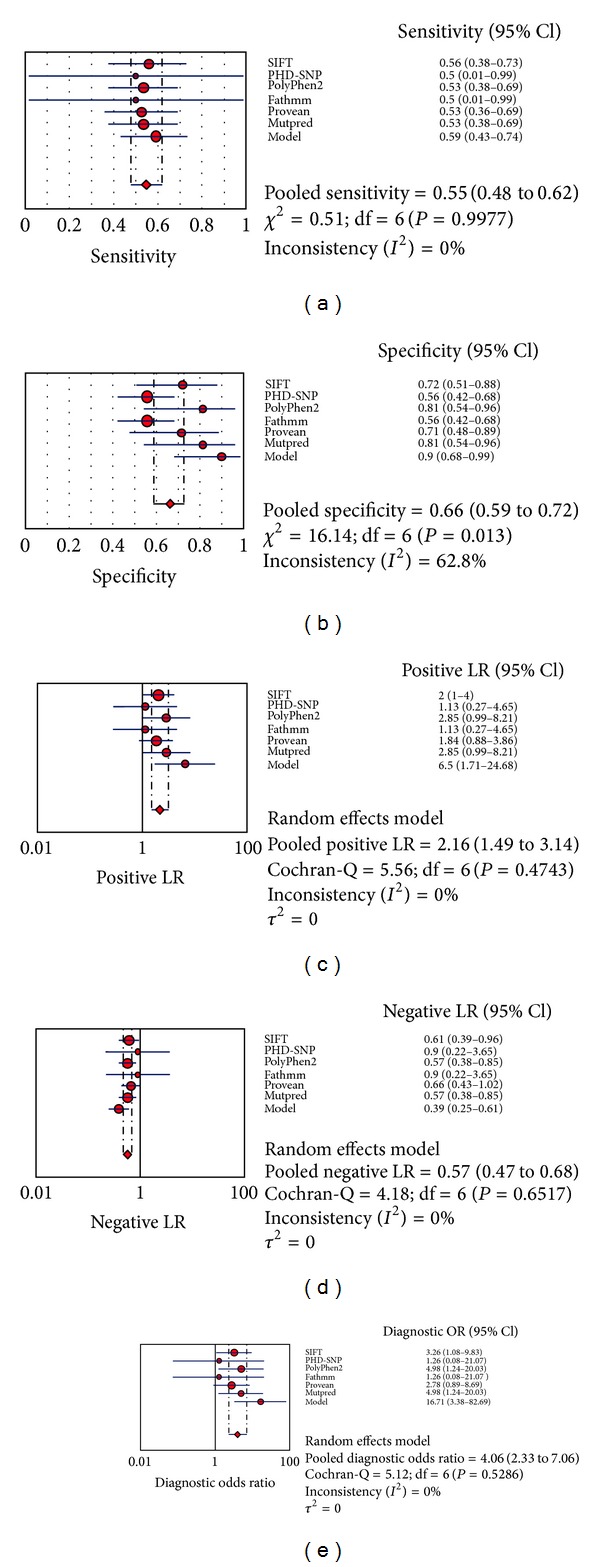
The individual and pooled diagnostic OR, sensitivity, specificity, negative likelihood ratio, positive likelihood ratio.

**Figure 3:**
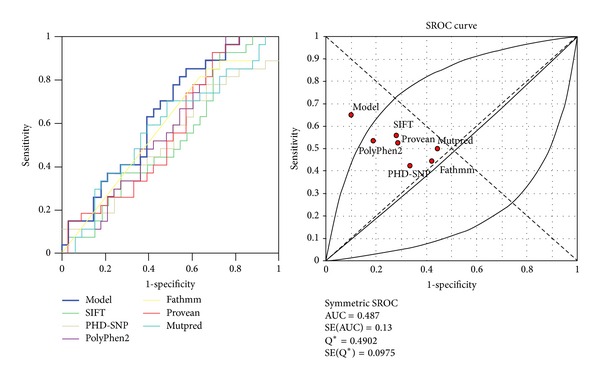
The summary receiver operating characteristic (SROC) curve of the selected SNP-based pathogenicity detection tools.

Overall, 59 different SNPs related to missense mutations in the *UGT1A1* gene were reviewed using the designed protocol (Figure 1). Our regression model was as *y* = 3.39 + (−0.24 × functional score) + (−0.14 × GV score) + (−2.44 × conservation score). Comparing the diagnostic OR, our model showed high detection potential (diagnostic OR: 16.71, 95% CI: 3.38–82.69) (Figure 2). The highest MCC and ACC was belonged to our suggested model (46.8% and 73.3%), followed by SIFT (34.19% and 62.71%) (Table 2). The SROC curves reflected an acceptable and fairly good overall diagnostic performance for our suggested model compared to the SNP-based pathogenicity detection tools (Figure 3). The AUC analysis showed a significance overall performance of our suggested model compared to the selected SNP-based pathogenicity detection tool (Table 3). 

## 4. Discussion

Since the late 1990s, the initiation of research using genetic testing or molecular medicine, development of diagnostics accuracy tests, and molecular assays that measure levels of genes or specific mutations are used to provide a specific therapy for an individual's diseases. We suggested a regression model based on different scores including functional scores, conservation score, and genomic variation degree and compared the results to the published clinical result as reference. We observed the effect of a set of disease-causing missense mutations, determined from the general population. The susceptibility of Mendelian inherited disease is most frequently associated with SNPs; hence, the mechanisms by which this occurs are still poorly known. From a biological point of view, the mutated residues are important for the proper functioning of a suitable protein structure [28]. 

Genetic variation in phenotype of the diseases is often difficult to detect because of the complex genetic nature of these species. Using functional characteristics of the genetic mutation will provide a powerful tool to uncovering genetic traits in more complex species and provide novel insights into the molecular mechanisms of the diseases [29]. More importantly, the associations between genetic variations of SNPs of candidate genes that are selected to represent the phenotype are variable and an important feature from the disease study point of view [30].

Sensitivity was not reduced, while higher sensitivity was observed in our suggested model followed by PolyPhen2, Mutpred, and SIFT. We compared our suggested model to several well-established SNP-based pathogenicity detection tools, by which the satisfactory performance of our model and SIFT indicates the importance of a mutation position in the context of the entire protein. It is therefore reasonable to believe that analyzing the results of some SNP-based pathogenicity detection tools such as, our proposed model, SIFT and PolyPhen2 is both feasible and promising but not very excellent. 

Saunders and Baker [31] and Bao and Cui [32] claimed that in case of unavailability of the conservation score, structural characteristics are valuable predictors. In this study we support using the sequence conservation score which is a good predictor and showed that an acceptable level of accuracy is achieved using the conservation score. Dobson et al., used machine learning methods to measure the sequence conservation score and showed that it is the most powerful single predictor and reported a high level of accuracy using the conservation score alone [33]. They also reported higher accuracy in structural characteristics in combination with the conservation score. We also showed that structural characteristics in combination with the conservation score improves prediction accuracy and can reduce the error rate of the conservation score alone. 

Ng and Henikoff used sequence and/or structure to predict the effect of a missense mutation on protein function in a mathematical model and claimed that their suggested model is a good SNP-based pathogenicity detection tools [13]. Capriotti et al. [14] developed a mathematical method that started from the protein sequence information, which can predict whether a new phenotype derived from a nsSNP can be related to a genetic disease in humans. They reported more than 74% accuracy in predicting whether a single point mutation can be disease related or not. Stitziel et al. [15] introduced a tool based on the hidden Markov models (HMM) for analyzing sequence homology of SNPs and reported 68% accuracy in predicting whether a single point mutation can be disease related or not. Shihab et al. [16], described a functional analysis Through Hidden markov models software and server and reported 71% accuracy in the predicton, which was less than SIFT (74%) but equal to PolyPhen2 (71%). Choi et al. [17] developed a new algorithm, which provides a generalized approach to predict the functional effects of protein sequence variations including single or multiple amino acid substitutions and in-frame insertions and deletions. They reported 84.8% accuracy compared to SIFT (84.5%) and PolyPhen2 (84.7%) in whether predicting that mutation can be disease related or not. In the present study we observed the highest accuracy with our suggested model as 73.33% compared with SIFT (62.71%) followed by PolyPhen2 and Mutpred (61.02%, in both). 

## 5. Conclusions

Our suggested model is comparable to the well-established SNP-based pathogenicity detection tools and can appropriately reflect the role of a disease-associated SNP in both local and global structures. A major drawback of the weighted SNP-based pathogenicity detection tools is the inherited restriction that falls within conserved protein domains. Hence, unlike other sequence-based prediction tools, which are too slow for practical use in large-scale sequencing projects, the weighted tools are computationally inexpensive and fast. Although the accuracy of our suggested model is not relatively high, the functional impact of the pathogenic mutations at the protein level is highlighted, which improves the understanding of the molecular basis of mutation pathogenesis.

## Figures and Tables

**Table 1:** Prediction results of SNP-based pathogenicity detection tools compared with the published results.

0	SNP-ID	Variant	SIFT	PhD-SNP	PolyPhen-2	fathmm	Provean	MutPred	References
(1)	rs74720349	V3G	1	0	0	0	0	1	0
(2)	rs201984525	L11P	0	1	0	0	0	1	0
(3)	rs111033541	L15R	1	1	1	0	1	1	1
(4)	rs72551339	H39D	1	1	1	1	1	1	1
(5)	rs140365717	E56A	1	0	1	1	1	1	0
(6)	rs4148323	G71R	0	1	1	1	0	0	1
(7)	rs72551340	F83I	0	1	0	1	1	0	1
(8)	rs144217005	V109A	0	1	0	1	0	0	0
(9)	rs140867457	I116K	0	0	0	1	1	0	0
(10)	rs200734586	K118N	0	0	0	1	0	1	0
(11)	rs72551341	L175Q	1	0	1	1	1	1	1
(12)	rs72551342	C177R	0	0	1	1	1	1	1
(13)	rs201093245	Y192C	1	0	1	1	1	1	0
(14)	rs72551343	R209W	1	0	1	1	1	1	1
(15)	rs144398951	I215V	0	0	0	1	0	0	0
(16)	rs144721642	V225M	0	0	0	1	0	0	0
(17)	rs35003977	V225G	0	0	0	1	1	1	1
(18)	rs35350960	P229Q	0	1	1	1	0	1	1
(19)	rs147640261	T232N	0	0	0	1	0	1	0
(20)	rs57307513	S250P	0	1	0	1	0	1	0
(21)	rs141950052	P267R	1	1	1	1	1	1	0
(22)	rs143072292	V273F	1	1	0	1	1	1	0
(23)	rs72551345	G276R	1	1	1	1	1	1	1
(24)	rs72551347	I294T	1	0	1	1	1	1	1
(25)	rs62625011	G308E	1	1	1	1	1	1	1
(26)	rs114000345	K317E	0	1	0	0	0	0	0
(27)	rs200903749	I322V	1	0	1	1	0	1	1
(28)	rs17851756	I322T	1	1	1	1	1	1	0
(29)	rs202035422	I329T	1	0	1	1	1	1	1
(30)	rs72551348	Q331R	1	1	1	1	1	1	1
(31)	rs139607673	R336W	1	1	1	1	1	1	1
(32)	rs144978321	S343L	0	1	1	1	1	1	0
(33)	rs149750520	N344K	1	0	1	1	1	0	0
(34)	rs201372184	A346V	1	1	1	1	0	1	1
(35)	rs72551351	Q357R	1	1	1	1	1	1	1
(36)	rs34946978	P364L	0	0	1	1	1	0	0
(37)	rs55750087	R367G	1	0	1	1	1	1	1
(38)	rs72551352	A368T	1	0	1	1	1	1	1
(39)	rs72551353	S375F	1	0	1	1	1	1	1
(40)	rs72551354	S381R	1	1	0	1	0	1	1
(41)	rs143573365	V386I	0	0	1	1	0	0	0
(42)	rs28934877	N400H	1	1	1	1	1	1	1
(43)	rs72551355	A401P	0	1	1	1	1	1	1
(44)	rs140613392	R403H	0	0	1	1	1	1	0
(45)	rs36076514	V411L	0	0	0	1	0	1	0
(46)	rs72551356	K428E	0	0	1	1	1	1	1
(47)	rs202172337	M441T	0	0	0	1	0	0	0
(48)	rs143033456	R442C	1	1	1	1	1	0	0
(49)	rs201427749	R450C	1	0	1	1	1	0	0
(50)	rs200370335	R450H	1	0	1	1	1	1	0
(51)	rs114982090	P451L	1	1	1	1	1	1	0
(52)	rs115410088	F460L	1	0	1	1	1	1	0
(53)	rs72551358	E463A	1	1	1	1	1	1	0
(54)	rs115944950	E463D	0	1	1	1	0	1	0
(55)	rs72551359	L474M	1	1	1	1	0	0	0
(56)	rs150687296	R475H	1	0	1	1	1	1	0
(57)	rs34993780	S488C	0	1	1	1	1	1	0
(58)	rs72551360	V499M	1	0	1	1	0	0	1
(59)	rs199723856	A511P	0	1	1	1	0	1	0

Disease: 1; neutral: 0; references: OMIM, PMID, SNPdbe, HGMD, and Swissvar results.

**Table 2:** Calculated Matthew's correlation coefficient (MCC) and accuracy (ACC) of the selected SNP-based pathogenicity detection tools and suggested model.

Detection tools	TP	FP	FN	TN	MCC	ACC
SIFT	19	7	15	18	34.19%	62.71%
PHD-SNP	1	27	1	34	3.39%	55.56%
PolyPhen2	23	3	20	13	29.89%	61.02%
fathmm	1	27	1	34	3.39%	55.56%
Provean	20	6	18	15	29.99%	59.32%
MutPred	23	3	20	13	29.89%	61.02%
Model	26	2	14	18	46.80%	73.33%

TP: true positive; TN: true negative; FP: false positive; FN: false negative; MCC: Matthew's correlation coefficient; ACC: accuracy.

**Table 3:** Area under curve for all the selected SNP-based pathogenicity detection tools.

		Area under the curve			
Tools	Area	Std. error	*P* value	95% Confidence Interval	
Model	.639	.071	.046	.499	.778
SIFT	.527	.075	.716	.380	.675
PolyPhen2	.516	.076	.829	.367	.666
PHD-SNP	.571	.074	.345	.426	.716
Provean	.587	.074	.249	.442	.732
Fathmm	.560	.075	.427	.413	.707
Mutpred	.580	.075	.288	.433	.727

*Significant, *P* < .05.
